# Is partnership the answer? Delivering the national immunisation programme in the new English health system: a mixed methods study

**DOI:** 10.1186/s12889-019-6400-6

**Published:** 2019-01-17

**Authors:** Tracey Chantler, Sadie Bell, Vanessa Saliba, Catherine Heffernan, Thara Raj, Mary Ramsay, Sandra Mounier-Jack

**Affiliations:** 1 London School of Hygiene & Tropical Medicine, Faculty of Public Health & Policy, 15-17 Tavistock Place, London, WC1H 9SH England; 2 Immunisation, Hepatitis & Blood Safety Department, National Infection Service, Public Health England, 61 Colindale Avenue, London, England; 3 NHS England (London Region), 5th Floor, Skipton House, 80 London Road, London, SE1 6LH England; 4 Bristol City Council, Public Health, Bristol City Council, City Hall (formerly The Council House), College Green, Bristol, BS1 5TR England

**Keywords:** Immunisation, Partnership, Collaboration, Mixed methods, Survey, Qualitative research, Health system reorganisation, Public health, Health systems, Health services

## Abstract

**Background:**

The English national health system experienced a major reorganisation in April 2013. This mixed methods study examined how staff managed to deliver the national immunisation programme within a new health infrastructure and explored the role and contribution of ‘partnership working’ to programme implementation.

**Methods:**

A cross-sectional online questionnaire survey and a qualitative evaluation of an urban immunisation board were conducted in 2016. The questionnaire included 38 questions about immunisation responsibilities, collaboration, service evaluation and programme support. It was completed by 199 immunisation providers and 70 people involved in the management of the immunisation programme. The evaluation involved 12 semi-structured interviews, 3 observations of forum meetings and the review of forum meeting minutes. Descriptive statistical analysis of the survey data was performed using SPSS version 23 and qualitative data from both study components were uploaded to NVivo 11 and analysed thematically.

**Results:**

Screening and Immunisation Teams were cited as responsible for programme leadership by 56% of survey respondents, but concerns were raised about their capacity to oversee larger geographies and a case made for decentralised accountability mechanisms. Only 44% of immunisation managers stated that poor performance was addressed adequately, and half of respondents thought that support given to providers was inadequate. Managers reported that partnership working improved the organisation (83%) and performance (78%) of immunisation, but stated it was more beneficial for information-sharing than implementation. A preference for a *“locality working approach”* with committees covering smaller health economies rather than larger commissioning areas was voiced. The immunisation board examined in the qualitative evaluation sought to achieve this by forging links with locally based steering committees, but also had to address internal challenges related to the role of the board and contribution of members to programmatic decision-making.

**Conclusions:**

Key challenges in delivering the immunisation programme were rooted in the new health infrastructure, which had created greater distance between commissioners and providers and resulted in the fragmentation of programme responsibilities. Partnership working bridged gaps but more needs to be done to strengthen accountability mechanisms and ensure that collaborative activities are outcome oriented and sustainable in the shifting environment of reorganisation.

**Electronic supplementary material:**

The online version of this article (10.1186/s12889-019-6400-6) contains supplementary material, which is available to authorized users.

## Key study conclusions and limitations

### Survey

- The distribution of roles and responsibilities for the English national immunisation programme across health organisations remained unclear three years after the April 2013 NHS reforms.
- The 2013 reformed commissioning footprint limited interaction between immunisation ‘managers’ and ‘providers’. A case was made for a more decentralised approach to performance management and training.
- Partnership working facilitated information sharing, but was less effective at promoting shared action and hindered by budgetary constraints.
- Key features of effective partnerships are trusting relationships, being able to build on existing working ties and workforce continuity.
- Despite these constraints most survey respondents had confidence in the system for delivering the immunisation programme in their area.
- The response rate means the sample is unlikely to be representative of all immunisation managers and service providers in England.

### Qualitative evaluation of the metropolitan immunisation board

- These types of partnerships need to maintain a strategic edge and be transparent about members’ roles in programmatic decision-making.
- Fostering collective affiliation was viewed as key to achieving mutually beneficial public health outcomes.
- Members were more actively engaged if the agenda was directly related to their professional responsibilities.
- Although the findings are not generalizable the in-depth analysis led to changes in the board’s terms of reference that are being implemented.

## Background

Effective partnership working between and within organisations was deemed an essential ingredient of Andrew Lansley’s vision for the new public health system in England [1]. Despite the emphasis placed on partnership working in public health over the past decades, it has proven difficult to measure its contribution to achieving health outcomes [2–4]. This paucity of evidence reflects the complex configuration of partnerships, their focus on process over outcomes, and the difficulty of measuring the impact of collaboration. It is also important to note that English public health partnerships have been hampered by repeated health system restructuring [5, 6].

Immunisation provides an interesting example of the role partnerships can play in managing public health programmes. In a preceding study we found that ‘partnership work’ was critical in the reintegration of the immunisation programme after the major reorganisation of the English national health system (NHS) in April 2013 [7]. This reorganisation was triggered by the Lansley’s 2012 Health and Social Care Act [8] and resulted in significant changes to delivery of the immunisation programme and the delegation of responsibilities.

### Changes to the system for delivering the immunisation programme

These changes are reported in full in our preceding paper and summarised here and in Table 1. NHS Primary Care Trusts, which were previously solely responsible for the local commissioning, coordination and evaluation of immunisation programmes, were abolished in April 2013 and their responsibilities distributed across different organisations. Screening and Immunisation Teams led by Public Health England were embedded within NHS England commissioning organisations and delegated responsibility for managing the delivery of the immunisation programme across larger areas. New General Practitioner led Clinical Commissioning Groups were delegated responsibility for overseeing quality improvement in primary care (delivery point for most immunisation programmes), and newly formed Local Authority Public Health Teams given responsibility for scrutinising the delivery of immunisation programmes.

**Table 1:** National immunisation programme infrastructure pre and post NHS re-organisation 2013 Source: This table was adapted from information contained in the Immunisation and Screening National Delivery Framework & Local Operating Model [9] & BioMed Central was the original publisher [7]

Key system component	Responsible organisation – pre-reform	Responsible organisation – post reform (April 2013)
Policy development, advice to ministers	Department of Health (national)	Department of Health (national)
Vaccine Procurement	Department of Health (national)	Public Health England (national)
Commissioning	Primary Care Trust (local)	NHS England (national)
• 16 national programmes		Local authorities (local) or NHS England (national)
• School based programmes		
Disease surveillance/ Outbreak response	Health Protection Agency (national)	Public Health England (national) and NHS England (national)
Advocacy, communication and health promotion	Primary Care Trust (local)	Public Health England (national)
		Local authorities (local)
System coordination	Primary Care Trust (local)	NHS England (national)
Vaccine, Cold Chain and Logistics Management	Primary Care Trust (local)	• Public Health England (national)
Vaccine Delivery	General Practitioners (local), NHS Community Trusts (local), other providers (local or national)	General Practitioners (local), NHS Community Trusts (local), other providers (local or national)
Child Health Information System (CHIS) and Data management	Primary Care Trusts through Child Health Information Systems (local)	Child Health Departments through Child Health Information Systems (local)
Workforce training	Primary Care Trusts (local)	Health Education England (national)
Others: Needs assessments, scrutiny and system assurance.	• Primary Care Trusts (local)	Local Authorities (local)
Others: Quality improvement (Duty of)	• Primary Care Trusts (local)	Clinical Commissioning Groups (local but need to give assurance to NHS England which is national)

It is well documented that large-scale health system reorganisations disrupt and obscure accountabilities for public health functions, and have detrimental effects on the well-being of staff [10–13]. There is less evidence on how staff manage to adapt and deliver population based immunisation programmes within new health systems. Our research seeks to address this gap in the literature. This study was part of a longitudinal analysis (2014–2017) of the effects of the April 2013 NHS reorganisation on the national population level immunisation programme. Preceding research applied a qualitative case study methodology to investigate the management and effects of change at national level and in three implementation sites in different regions of England. In this mixed methods study conducted in 2016 we sought to evaluate the transferability of the findings generated from the pre-ceding research across more implementation sites in England. Specifically, we were interested in gaining more insights into how partnership working was helping to streamline and reintegrate the delivery of the immunisation programme following the fragmentation, which was a by-product of the 2013 NHS reorganisation. To achieve this we conducted a cross-sectional survey targeted at immunisation ‘managers’ and ‘providers’ in England, and a qualitative evaluation of a metropolitan immunisation board that was seeking to renew its terms of reference three years after it was formed in 2013. The findings provide useful insights into collaborative practice across organisations and highlight system vulnerabilities that could have a bearing on immunisation performance.

## Methods

Between June–September 2016 we conducted a cross-sectional questionnaire survey targeted at immunisation ‘managers’ and service ‘providers’ in England, and a qualitative evaluation of the terms of reference of an immunisation board that served a large metropolitan area. The methods we used for each are presented separately.

### Cross-sectional questionnaire survey

#### Sample

The sampling frame consisted of ‘providers’ i.e. those responsible for delivering the immunisation programme in general practice or in community care settings, and ‘managers’ i.e. those who commission, manage or play a role in service quality improvement or population level health protection. This included NHS England and Clinical Commissioning Group (CCG) commissioners and members of Screening and Immunisation Teams (SIT), Health Protection Teams (HPT) and local authority public health teams (LA PHT).

#### Instrument

The questionnaire (Additional file 1) was informed by the findings of the preceding study [7] and arranged into four sections: ‘Professional demographics’, ‘Individual and organisational responsibility for immunisation’, ‘Working with others to manage the immunisation programme’, and ‘Evaluating programme uptake and service quality’. It comprised of 38 questions and took 20–30 min to complete; response options included Likert scales, multiple choice, binary data and free text. The online questionnaire and distribution platform were tested internally and piloted with eight participants from the preceding study [7].

#### Procedure

Three strategies were used to target the sample group; i) we sent 850 invitation and reminder emails with survey web links to named immunisation managers identified with the support of colleagues from Public Health England (PHE) and NHS England and by checking relevant websites, ii) we invited 70–80 members of a regional immunisation network by sending a generic invitation with anonymous survey web-link, iii) the survey was advertised with the anonymous web-link in monthly vaccine update newsletters sent to 38,500 subscribers. Respondents who were willing to complete the questionnaire were asked to tick a Yes box at the start of the questionnaire to indicate that they consented to take part in the study.

A total of 104 questionnaires were received via the online platform which equated to a response rate of 13.8% after the target number reduced to 753 because 97 emails bounced. Accurate response rates could not be calculated for the other strategies which resulted in the return of 260 questionnaires. Of the 364 questionnaires received, 185 (51%) were fully completed. Incomplete questionnaires were reviewed and only those with information entered beyond section one (professional demographics) (*n* = 278, 76%) were included in the analysis.

#### Analysis

Frequency and percentage distributions were used to summarise closed text responses. In the results section, alongside percentages, the numerator and denominator for responses to survey questions are recorded for clarity and to highlight incomplete data and survey drop out, which is evident when the denominator decreases. Responses were examined collectively, by NHS region (London; South of England; Midlands or East of England; North of England), and then separately for managers and providers. It was not possible to draw inferences from the data based on professional roles or NHS regions due to the low number of survey responses when explored by these variables. For closed responses, data management and analysis was performed using SPSS version 23. Open text responses were downloaded to NVivo 11 (qualitative data analysis software) and analysed deductively [14] to add descriptive value to the quantitative data.

### Qualitative evaluation of a partnership forum

As described (Text Box 2, Table 1) the enactment of the 2012 Health and Social Care Act (HSCA) on 1st April 2013 led to significant changes in the structure and organisation of the English health system. To manage these changes NHS England and PHE immunisation leads based in a large metropolitan area established an Immunisation Board in November 2013. The goal was to create a partnership forum that would; i) clarify responsibilities and coordinate efforts across organisations, ii)provide oversight of the delivery of the immunisation programme and activities aimed at increasing vaccination coverage, and iii) provide a means of organisational accountability. In March 2016 the leadership of this immunisation board, decided to renew the board’s terms of reference (TOR) to ensure the board remained fit for purpose. To inform TOR revisions LSHTM researchers were asked to conduct a qualitative evaluation of the immunisation board to document members’ perspectives on: i) its purpose, ii) its governance, iii) its achievements (public health outcomes), iv) members roles and responsibilities, v) operational challenges and vi) whether meeting arrangements (e.g. schedule, communication) facilitated pragmatic partnership work.

The methods applied in this evaluation were semi-structured interviews with board members, observations of board meetings and a review of board meeting minutes.

#### Data collection and analysis

Data was collected from June–September 2016. The sample frame was the board membership list provided to LSHTM researchers in May 2016 which listed 26 people. These were lay members and representatives from the following organisations: NHS England, PHE, Universities, CCGs, Local Councils (authorities/boroughs) and service provider organisations.

Three members were excluded from this sample because they had been involved in planning this study. The remaining 23 received an email and follow-up phone call inviting them to participate in a semi-structured interview. Twelve responded favourably, eight did not reply, two declined participation since they had not attended recent meetings, and one email bounced. The review of 2014–15 board minutes indicated that six non-respondents had either not attended or attended less than one board meeting in that time-period.

Nine interviews were conducted in person by a LSHTM researcher in places of participants choosing (place of work, café, or LSHTM) and three by phone. Prior to the interviews, the purpose of the study was discussed and interviewees signed a consent form stating willingness to participate. The interviews lasted 20–60 min, were recorded with participants’ permission, transcribed and uploaded to NVivo 11. The approach to data analysis was thematic and involved a combination of deductive and inductive coding [14]. This consisted of organising the data under the pre-defined topic areas (topic guide in Additional file 2) from the interview guide and then exploring this data inductively to identify the key themes and associated sub-points.

## Results

The results for each study method are presented separately. Key and complementary findings are synthesized in the discussion with reference to relevant literature.

### Cross-sectional questionnaire

#### Questionnaire sample characteristics

The sample included 278 respondents, of which 72% (*n* = 199) were providers and 28% (*n* = 79) were managers. Of the providers 156 worked in General Practice and the rest were involved in the provision of immunisation in community based settings e.g. schools. All four NHS England regions were represented across both groups although there were more provider respondents in the South of England and fewer manager respondents in the Midlands and East of England (Table 2).

**Table 2:** Survey respondents

Role	Region				
	London	South of England	Midlands or East of England	North of England	Total
Managers	23	23	12	21	79
Providers	46	58	50	45	199
Total	69	81	62	66	278

#### Roles and responsibilities for immunisation

The reorganisation of the NHS in April 2013 was reported to have resulted in a lack of clarity about organisational roles and responsibilities for immunisation, and led to duplication of work.

*“I feel having so many organisations involved initiates blurred lines of responsibility, often causing delayed action and leaving the staff on the shop floor uninformed and unprepared.”* Practice nurse, Midlands or East of England

Most managers (84% *n* = 66/79) stated that the SITs were responsible for programme leadership, although concerns were raised about their capacity to oversee new larger areas. London managers were least likely to identify SITs as the programme leads (61% *n* = 14/23), and South of England managers most likely (96% *n* = 22/23). Providers were less certain about programme leadership, only 46% (*n* = 89/197) identified the SITs as the leads, 28% (*n* = 56/197) selected an organisation other than SITs and 26% (*n* = 52/197) were unsure. With regards to more specific roles managers were very confident about who was responsible for commissioning the immunisation programme, but much less clear about whose role it was to provide training, and monitor the quality of service provision (Table 3). Providers were clearer about who was responsible for conducting training, but less sure about who was responsible for evaluating the quality of immunisation activities.

**Table 3:** Proportion of respondents who were clear about the delegation of responsibilities for immunisation

Questionnaire statement	Managers by region					Providers by region				
	London (*n* = 22)	South of England (*n* = 23)	Midlands or East of England (*n* = 12)	North of England (*n* = 20)	Total (*n* = 77)	London (*n* = 43)	South of England (*n* = 55)	Midlands or East of England (*n* = 46)	North of England (*n* = 44)	Total (*n* = 188)
The organisation responsible for commissioning the immunisation programme	86% (*n* = 19)	91% (*n* = 21)	92% (*n* = 11)	95% (*n* = 19)	91% (*n* = 70)	58% (*n* = 25)	46% (*n* = 25)	57% (*n* = 26)	59% (*n* = 26)	54 (*n* = 102)
The organisation responsible for training immunisation providers	27% (*n* = 6)	26% (*n* = 6)	42% (*n* = 5)	35% (*n* = 7)	31% (*n* = 24)	51% (*n* = 22)	47% (*n* = 26)	41% (*n* = 19)	75.0% (*n* = 33)	53% (*n* = 100)
The organisation responsible for monitoring and evaluating the quality of service providers’ immunisation activities	50% (*n* = 11)	70% (*n* = 16)	67% (*n* = 8)	70% (*n* = 14)	64% (*n* = 39)	47% (*n* = 20)	35% (*n* = 19)	41% (*n* = 19)	59% (*n* = 26)	45% (*n* = 84)
The distribution of roles and responsibilities for immunisation across different organisations	23% (*n* = 5)	48% (*n* = 11)	67% (*n* = 8)	30% (*n* = 6)	39% (*n* = 30)	35% (*n* = 15)	27% (*n* = 15)	35% (*n* = 16)	57% (*n* = 25)	37.8% (*n* = 71)

#### Partnership working

For this section, only data from managers was analysed since individual providers were less involved in partnership working. Almost all managers (96%; n = 72/75) attended at least one public health partnership body, in which immunisation was discussed, on a quarterly or less regular basis. These partnership forums included Health and Well-being Boards^1^ and Health Protection Forums^2^ established as part of the health system reorganisation, CCG influenza meetings, and immunisation committees that formed more organically. More managers were involved in local immunisation committees (77% *n* = 58/75) than Health Protection Forums (61% *n* = 46/75) or Health and Well-being Boards (53% *n* = 40/75). Immunisation committees varied in geographic scope (wider commissioning areas or smaller CCG or LA geographies), subject focus (health protection/resilience, the whole immunisation programme or specific vaccine services e.g. flu), the attendance (some or all partners), who chairs the committee, and the remit (strategic oversight or operational input). In free text comments respondents expressed preference for a *“locality working approach”* with committees covering smaller health economies (e.g. LAs or CCGs) rather than larger commissioning areas.

*“The PHE screening and immunisation team cover too wide an area for all areas to be involved in their committees and therefore have little impact upon local area issues.”* Deputy Director of Public Health, North of England

#### The benefits of partnership

Most managers reported that partnership working was beneficial to improving the organisation (83% *n* = 60/72) and performance (78% *n* = 56/72) of the immunisation programme in their area. London respondents were most positive about how partnership working strengthened collaborative activities listed in Table 4. Across all regions, managers were more likely to report partnership working as beneficial to the broader sharing of information and knowledge, than to the delegation of responsibilities, addressing poor performance and improving programme implementation (e.g. provision of training).

**Table 4:** Proportion (%) of managers who *agreed (fairly or very strongly)* that partnership working helped with specific activities

Activities	*n* = 72
Information sharing	85% (61)
Sharing provider uptake data	83% (60)
Sharing expertise across organisations	76% (55)
Joint planning on how to increase coverage	76% (55)
Understanding local barriers	74% (53)
Local authority scrutiny of immunisation performance	69% (50)
Agreeing local priorities, setting targets and delegating responsibilities	58% (42)
Increasing access to immunisation services for people living in your area	58% (42)
Clarifying different organisations’ roles and responsibilities	57% (41)
Addressing poor performance at provider level	47% (34)
Organising training and support to providers	36% (26)

#### Barriers and enables to partnership working

The main reported barriers to partnership working were: 1) lack of budget 72% (n = 52/72), 2) lack of shared ownership 69% (*n* = 50/72) and 3) lack of commitment from one or more partners 65% (*n* = 47/72). The lack of strategic leadership was highlighted as a very significant barrier for 35% (*n* = 25/72) and a fairly significant one for 22% (*n* = 16/72). Other barriers cited in free text included the larger footprint of commissioning areas, the complexity of the new health system, the lack of geographical co-terminosity between partner organisations and the loss of professional and local knowledge during the health system reorganisation.

When asked for facilitators of partnership working (the factors that supported the development of partnerships), respondents stressed the importance of building on existing working relationships (“*Historical links between CCG and local authority teams - originally both NHS - promote good working relationships.”* Senior Public Health Specialist, London) and fostering new networks. Continuity of staff and ensuring the visibility of SITs on the ground, (by giving them manageable geographical areas of responsibility), were also viewed as critical to building strong partnerships. Other facilitating factors highlighted were face-to-face meetings, open and honest dialogue, clarity about roles and responsibilities, identifying concrete issues (e.g. low uptake) that require joint action, being able to access resources and adopting a *“localised approach”* of working. Over a third of managers also thought that they would benefit from training in partnership working.

#### Supporting providers and evaluating programme performance

The support given to providers was considered adequate by 51% (*n* = 35/69) of managers and 55% (*n* = 68/124) of providers. Addressing poor performance was considered even more challenging with only 44% (*n* = 30/68) of managers stating this was adequate. Managers in London were more likely to assess this as inadequate (65%, *n* = 13/20) in comparison to other regions where rates ranged from 21 to 46%.

Most managers reported that SITs were very or quite involved in: supporting providers (81%; *n* = 57/70), evaluating their performance (93%; *n* = 65/70), and taking action to improve their performance (90%; *n* = 63/70). In contrast, less than half of managers reported that CCGs and LA-PHTs were very or quite involved in these activities.

Most providers had access to training updates but over a quarter stated that they did not think that foundation immunisation training was available (*n* = 34/124). Access to technical guidance was highly appreciated but concerns were raised about SITs capacity to cover large areas, and a case was made for more decentralised support and accountability mechanisms.

“*Devolve more responsibility to local authority teams in conjunction with CCGs to plan, deliver and challenge better”* Deputy Director of Public Health, North of England

Providers reported low level of visits by SITs (19%, *n* = 23/124) and even less from CCG members (10%, *n* = 12/124). They also reported that SITs positioning within NHS England and their dual role of providing technical guidance and leading on commissioning, meant that providers were less likely to seek their support.

*“Working for a provider it is noticeable that there is reluctance to engage with NHS England staff for advice and support. They are very much seen as the commissioners not the advisors… I am reprimanded for asking them for advice without first going through our business management.”* Immunisation coordinator for a provider organisation, North of England

#### Overall confidence in the system for delivering the immunisation programme

Despite some lack of clarity surrounding the roles and responsibilities of organisations in immunisation programme, most respondents had confidence in the system for delivering the immunisation programme in their area. However, it was evident that respondents were less confident about their local systems’ ability to deliver equitable services and address inequalities in performance. There were regional variations in responses, with the lowest confidence levels in London (Table 5). Overall providers expressed more confidence in the system for delivering the immunisation programme than managers.

**Table 5:** Proportion of respondents who had confidence in the overall immunisation system and its ability to deliver equitable and high quality services

Questionnaire statement	Managers					Providers				
	London	South of England	Midlands or East of England	North of England	Total	London	South of England	Midlands or East of England	North of England	Total
I have confidence in the system for delivering the immunisation programme in my area	63%	89%	82%	78%	78%	77%	86%	78%	93%	84%
	(*n* = 12/19)	(*n* = 17/19)	(*n* = 9/11)	(*n* = 14/18)	(*n* = 52/67)	(*n* = 24/31)	(*n* = 25/29)	(*n* = 21/27)	(*n* = 28/30)	(*n* = 98/117)
I have confidence that immunisation services in my area are delivered equitably	47%	63%	55%	61%	57%	72%	76%	73%	90%	78%
	(n = 9/19)	(n = 12/19)	(n = 6/11)	(n = 11/18)	(*n* = 38/67)	(n = 21/29)	(n = 22/29)	(n = 19/26)	(n = 26/29)	(*n* = 88/113)
I have confidence in the way that inequalities in performance are monitored and addressed in my area	33%	56%	44%	50%	46%	68%	52%	43%	61%	57%
	(n = 6/18)	(n = 10/18)	(n = 4/9)	(n = 9/18)	(*n* = 29/63)	(n = 19/28)	(n = 14/27)	(*n* = 10/23)	(n = 17/28)	(n = 60/106)

### Qualitative evaluation

Twelve board members were interviewed (Table 6) and 2 board meetings were observed. The data analysis distilled three overarching themes (‘Defining the board’s purpose and decision-making role’, ‘Promoting collective affiliation for mutually beneficial public health gains’, ‘Achievements, maintaining momentum and moving forward’), which are summarised below.

**Table 6:** Organisational affiliation of interviewees

Organisation affiliation	N
NHS England	3
Public Health England	2
Academia	1
Lay person	1
CCG	2
Provider	1
Local Authority Public Health Team	1
Local Councils	1
Total	12

#### Defining the board’s purpose and decision-making role

The term used most often to describe the purpose of the board was *‘oversight’*. Interviewees considered the board responsible for overseeing commissioning and providing input into commissioning decisions. The nature of this board was less clear, to paraphrase: *‘Is it a steering committee, a partnership forum, or a formal decision-making structure?’*

Interviewees thought they could contribute valuable insights into decision-making, but it was argued that the role of the board in informing decision-making needed to be more transparent.

*“I think probably, what might be helpful is having clarity around what the board is being asked to do when papers come to them… I don’t think this a decision-making body, to my knowledge the decisions and the accountability sit with the people in the system rather than with the board…so being clear about what it is you’re asking people to guide and advise on, and coming back to them to say, “Well we did this, as a result of that”.”* Board member #11

Interviewees wanted the board to demonstrate more strategic leadership: *“…the board should be about providing the leadership and the direction and the assurance and the challenge, as well, around immunisation performance…the board should be absolutely on our backs constantly”* (Board member #2). It was also stated that it needed to do better at holding NHS England to account, and delivering agreed strategies, e.g. establishing borough level immunisation steering committees with local action plans.

#### Promoting collective affiliation for mutually beneficial public health gains

At functional level, some interviewees raised concerns about what they perceived to be a lack of collective affiliation and common goals.

*“You know, various people will be round the table and the impression … I can only tell you what my sense is, but it’s that people are there either to find out something or to bash the (lead organisation) with something, or to … you know, to lobby for their own agenda.”* Board member # 2

Not detracting from the mandated responsibilities of the lead organisation, a case was made for promoting more collective responsibility to achieve mutually beneficial public health gains.

The leadership wanted members to identify themselves as immunisation champions however not everyone agreed with this expectation. One interviewee expressly stated she was not on the board as a subject expert, but to represent her organisations’ point of view: *“…which is sometimes at odds, because what I’m there sometimes to do is to say, ‘Actually that’s all very interesting, but there are other priorities, don’t forget.’ So, it’s more about putting it into context.”* (Board member# 6). Membership meant different things to different representatives, for a few it was just a means to stay in the loop whereas for others it was a way to ensure that decision-making accounted for the realities on the ground and was evidence-based. Several interviewees argued for clearer guidance about what is expected from board members.

*“I think we need to revisit exactly what our membership is and what each person thinks they’re bringing to the group and what skill and expertise they’re contributing.”* Board member # 9

#### Achievements, maintaining momentum and moving forward

The implementation of a Measles Mums & Rubella (MMR) catch-up campaign straight after the 2013 NHS reorganisation was presented as a key achievement and confirmation of the utility of a metropolitan partnership structure that serves a whole NHS England commissioning region. Interviewees also credited the board with supporting communication between partners and providing backing for new operational procedures.

Board members with an active rather than a watching brief for immunisation found it easier to prioritise attendance since the meetings corresponded with their direct responsibilities. Members were motivated if they could see they were adding value: *“If it is just a show and tell, people will skip it…”* (Board member #3), but when plans are openly discussed and modified in meetings they felt more engaged.

## Discussion

Three years after the April 2013 health system reorganisation in England the lack of clarity about the distribution of roles and responsibilities for the national immunisation programme persisted. Questionnaire respondents from London and providers reported especially lower levels of understanding of the distribution of roles and responsibilities among organisations involved in immunisation activities. Core areas of concern for survey respondents were the evaluation of programme performance and the level of support extended to immunisation providers. The larger commissioning footprint introduced as part of the April 2013 health system reorganisation was viewed as a significant barrier to service evaluation and partnership working, and a case was made for a more decentralised approach.

Most managers were actively engaged in partnership working and viewed it as beneficial to the broader sharing of information and knowledge. It was deemed less effective for delegating responsibilities and implementing the programme. The main barriers to partnership were the lack of budget and the lack of shared ownership and commitment. Despite the limitations of partnership working, it is interesting to note that respondents reported high overall confidence in the system for delivering the immunisation programme in their area, showing a good degree of resilience of the re-organised immunisation system. However, they were less assured of its ability to deliver equitable services and address inequalities in performance.

The qualitative evaluation indicated that partnership forums can help support programme coordination and implementation on the ground. A notable achievement for the metropolitan board was the implementation of the MMR catch up campaign in 2013 and this board was also credited with supporting communication and providing backing for new operational procedures. However, declining attendance and engagement of board members over time was also noted and was the key impetus for the evaluation of the board’s TORs.

The findings demonstrated that members were less likely to be engaged if they only had a watching brief for immunisation, or thought their contribution was not accounted for in decision-making. To regain momentum interviewees stated the board’s purpose and member’s contribution to strategic decision-making need to be clarified. Identifying common goals was also perceived as vital to achieve mutually beneficial public health gains.

### Comparison with existing literature

Health system reorganisation is known to disrupt effective collaborative networks [5, 15] and require staff to expend significant effort to re-establishing partnerships and make sense of new organisational arrangements [7]. In this context, it is easy for partnership working to become a means in itself rather than a means to an end. Hunter et al. [6] argue that working across organisational boundaries in public health must be focussed on achieving health outcomes rather than on the process of partnership work. They acknowledge the lack of evidence for the former [2, 3], and attribute this to a tendency to over-engineer partnerships, prioritising structures and targets over a flexible and integrated response to service users’ needs [15]. To guard against this tendency, partnerships need to have a clear raison d’ệtre with strong leadership and a focus on outcomes, bring together the right partners who can contribute most and commit, invest in building trust and relationships, establish joint delivery mechanisms and clear lines of accountability, and maintain a flexible approach conducive to innovation. Higher level partnerships were portrayed as more likely to become bogged down in bureaucracy whereas frontline ones were more able to identify problems and mount a joint-response [6].

This literature resonates with results from our study on several levels. Firstly, the questionnaire findings suggested partnership working was most effective at supporting information sharing and planning as opposed to operationalising action such as increasing access to immunisation services, organising training and addressing poor performance. The former are critical precedent activities however without the latter their value diminishes. Secondly, SIT’s capacity to cover the larger health commissioning geographies created following the April 2013 reorganisation was questioned, and many respondents called for a more localised approach to partnership working and to service evaluation. Supporting local partnerships can probably be viewed as a mitigating strategy for bridging the distance between SITs and providers and addressing more challenging “wicked” public health issues, such as reducing inequalities of uptake. Thirdly, in line with previous research, respondents stressed the importance of fostering strong and trusting partnership relationships and valued continuity of staff and being able to build on former working ties [16, 17]. These factors also assisted the organisation of a regional catch-up campaign in the case study, which is evidence that higher level partnerships can take action.

A wide range of immunisation partnership forums were cited in the questionnaire including short term, operationally oriented flu committees, to more formal Health and Wellbeing Boards with their broader geographical scope and agenda. The prevalence and variety of partnership forums indicates that they fill a gap and contribute to joined-up local systems. However, it does not tell us much about the strengths and weaknesses of these different partnership forums or the way they help deliver outcomes. Other authors have argued that the most effective partnerships are those which are not imposed but “built from the bottom-up” and adapted according to local service needs [15]. This guidance will be important for the metropolitan immunisation board, as it works more closely with a network of steering committees that operate at local authority level.

A key barrier to partnership working reported in the questionnaire was the lack of an allocated budget. The potential for resource constraints to undermine partnership working in public health has been highlighted elsewhere [18, 19], and is poignant in the current climate of austerity and associated public service funding constraints England. Partnership working has been mandated without any recognition that it is not a cost neutral activity. It is also not a quick fix; time is an essential ingredient in establishing effective partnerships [18]. Attention also needs to be paid to the leaderships skills required to *‘operate horizontally and vertically across organisations and have the skill set to bring key people and organisations together in a nurturing and constructive manner’* [15], p.5. Over a third of managers in our survey stated they would value more training in partnership working and lack of strategic leadership was cited as a significant barrier to collaboration.

### Strengths and weaknesses of this study

This study provides valuable insights into how a wide range of health professionals from different regions in England were managing to deliver the national immunisation programme following the April 2013 health system reorganisation. It also examines the functioning of an immunisation board operating across a large metropolitan area. The combination of closed and open questions in the questionnaire allowed us to quantify responses and gave respondents the opportunity to elaborate on their experience. The main limitation of the questionnaire was the response rate which means that the sample is unlikely to be representative of all immunisation managers and providers. Some respondents were also self-selecting and may have been more interested in immunisation and know more about the system than those who did not respond. This could mean that the results underestimate the level of uncertainty about the leaderships and distribution of roles in the immunisation programme. Of note here is the fact that the ‘manager’ group included people with differing levels of direct input into programme management ranging from commissioning to programme assurance. Finally, the questions on partnership working were less relevant to those delivering the immunisation programme in primary care. This may explain survey incompletion and dropout by providers; the number of providers declines by 38% in comparing questions between the start and end of the survey (*n* = 199 to *n* = 124).

The main limitation of the qualitative evaluation was its focus on a single partnership forum that operated at a regional level. This means that the lessons are learned are not generalizable, although they are transferable to similar settings. The strength of this in-depth analysis was that it led to changes in the board’s TORs that are being implemented. Moving forward more research is warranted on how regional partnership bodies can work effectively with immunisation steering committees that cover smaller geographic areas and can more n be ‘hands on’ in terms of delivering outcomes.

### Implications

This study highlights key challenges confronted by those delivering the national immunisation programme in the new health system. Many of these were rooted in the April 2013 health infrastructure, which created greater distance between commissioners and providers and resulted in a fragmentation of programme responsibilities across organisations. The increase in the geographical commissioning footprint left providers feeling unsupported and managers concerned about their ability to address poor performance. The local operating model suggests that CCGs may be able to assist SITs in providing support to practices but also states that CCGs do not have a contractual duty to do this [9]. The lack of clarity about partner responsibilities for quality and performance improvement in these guidelines is of concern. Without advocating more structural changes, we think that there is a need for improved utilisation of different partner organisations’ strengths. LA PHTs and CCGs are responsible for smaller geographic areas than SITs and could be in a better position to strengthen outreach to under-vaccinated communities and review the performance of their constituent practices, respectively. We do however appreciate that this is not straightforward given differing views about the transfer of the public health function from the NHS to local authorities [20, 21], and the lack of organisational uniformity across CCGs [22].

This paper posed the question as to whether partnership working is the answer to supporting the effective delivery of the immunisation programme. The findings and related literature suggest it could help address programme vulnerabilities if partnership structures have clear objectives, are action and outcome oriented and appropriately resourced. Partnership work is not resource neutral and given the current budgetary constraints facing public health practice in England [23] it is critical that the outcomes of collaborative activities contribute to service efficiencies. There is also a need to consider how immunisation and related partnership work fit within the 2016/17–2020/21 NHS planning strategy, which mandates placed-based collaborative working to restore and maintain financial balance and deliver core access and quality standards for patients [24]. As part of this strategy NHS organisation and Local Councils were required to form ‘Sustainability and Transformation Partnerships’ (STPs) or Accountable Care Systems (ACSs) and produce five- year plans for the provision of specialised and primary care services in their respective areas. The purpose of these plans is to devise practical ways to improve NHS services and population health that reflect the needs of residents in each area and encourage collaboration across organisations with responsibilities for health and well-being [25]. As such, STPs and ACS may be potential mechanisms for facilitating and funding partnership working that aims to improve the performance of the immunisation programme. Finally, this study also shows that it is good practice for partnership forums to evaluate their terms of references every couple of years to ensure that they remain fit for purpose.

## Conclusions

Despite the challenge of delivering population-based immunisation programmes in a more complex health system, managers and providers remained confident in their local systems’ ability to provide immunisation services. Partnership working was valued by managers, who re-regrouped in forums of different natures and shapes to deliver the immunisation programme. The benefits of partnership working were however mostly seen at the level of sharing information rather than implementing programmatic activities. This means that partnership efforts did not succeed at meeting providers’ needs for additional support in improving vaccination performance. Providers remained unsure about the new architecture of the immunisation system and lacked understanding of the roles and responsibilities of key stakeholders’ organisations. With the construction of the new immunisation system as a partnership, the issue now seems to be less whether partnerships are useful but rather how to shape partnership infrastructures in a way that is outcome oriented, as well as cost-effective and sustainable in the shifting environment of further reorganisation.

## Additional files

Additional file 1: Questionnaire regarding the delivery of the immunisation programme (PDF 225 kb)

Additional file 2: Topics covered in the interviews in the qualitative evaluation (PDF 259 kb)

## Abbreviations

ACSs: Accountable Care Systems
CCG: Clinical Commission Group
GP: General Practitioner
HPT: Health Protection Teams
LA PHTs: Local Authority Public Health Teams
NHS: National Health System
PCT: Primary Care Trust
PHE: Public Health England
SIT: Screening and Immunisation Team
STPs: Sustainability and Transformation Partnerships

## Footnotes

1 Health and Well-being boards are formal committees within the local authorities which are charged with promoting greater integration and partnership between bodies from the NHS, public health and local government. They have a statutory duty, with clinical commissioning groups (CCGs), to produce a joint strategic needs assessment and a joint health and wellbeing strategy for their local population.

2 Health Protection Forums aim to maintain a strategic overview of health protection matters across local authority geographies, and provide assurance that arrangements in place to protect the health of residents are sufficiently robust and respond to local needs. The forums are chaired by Directors of Health and typically involve public health commissioning leads, CCG members, environmental health officers, screening and immunisation team leads and consultant in communicable disease control.
