# Supplementary material for: Is partnership the answer? Delivering the national immunisation programme in the new English health system: a mixed methods study
Source: BMC Public Health. 2019 Jan 17;19:83. doi: 10.1186/s12889-019-6400-6 (PMC6337826; doi:10.1186/s12889-019-6400-6)
Supplement: Supplementary file 1 — Questionnaire regarding the delivery of the immunisation programme (PDF 225 kb) [file 12889_2019_6400_MOESM1_ESM.pdf]

Questionnaire about the delivery of the immunisation programme

Share your views about the delivery of the immunisation programme
Thank you for considering taking part in this survey - your views count.

Purpose of the survey

This survey is part of a longer-term analysis of the way that the national immunisation programme is being managed and delivered in the new health system (post April 2013). It is being conducted by the 'Health Protection Research Unit (HPRU) in Immunisation', which includes researchers from the London School of Hygiene & Tropical Medicine (LSHTM) and Public Health England. The unit is funded by the National Institute of Health Research.

The purpose of this survey is to determine how the national immunisation programme (specifically Section 7a programmes) is being delivered across different areas, how partner organisations are working together, and what is being done to monitor and improve the performance of immunisation services (e.g. quality and uptake). The findings will be shared with those responsible for making strategic and programmatic decisions about the management and delivery of the Section 7a immunisation programmes.

Have your say
We wish to obtain a wide range of views in order to better understand what facilitates and hinders progress in managing and delivering the immunisation programme. This is your chance to tell us what is working well and to highlight areas where you feel more input is needed.   Confidentiality and anonymity   Responses to this survey are strictly confidential. No-one outside the research team will be able to see your completed questionnaire, or to identify your individual responses. When reporting survey results, care will be taken to ensure that no individual or organisation can be identified. Completing the questionnaire The questionnaire will take about 20-30 minutes to complete. There is a bar at the top of each page to help you gauge your progress. If possible, please try and complete the questionnaire in one sitting. If you have any problems in accessing, navigating or completing the questionnaire, please do not hesitate to contact us for assistance via the following email address: IMMS_Survey@lshtm.ac.uk.   Feedback We plan to disseminate the survey findings as widely as possible in journals and newsletters read by people involved in managing and delivering the immunisation programme in England. You can also ask to receive a summary of the findings, once these have been written up, by emailing us via the following email address: IMMS_Survey@lshtm.ac.uk

Tracey Chantler, Research Fellow, LSHTM,15-17 Tavistock Place, London, WC1H 9SH
tracey.chantler@lshtm.ac.uk or IMMS_Survey@lshtm.ac.uk

Sandra Mounier-Jack, Senior Lecturer, LSHTM, 15-17 Tavistock Place, London,WC1H 9SH
IMMS_Survey@lshtm.ac.uk

LSHTM Ethics Ref: 8661

Opt-in consent
Completing the survey is completely voluntary. You may receive a reminder but you are under no obligation to take part in this survey. If you are happy to take part in this survey, please tick Yes.

- ❍ Yes (1)

Section 1: Professional demographics
In this section you will be asked about your professional background and where you are based.

Q1 Which of the following four regions (as defined by NHS England) do you work in?

- ❍ London (1)
- ❍ South of England (2)
- ❍ Midlands or East of England (3)
- ❍ North of England (4)

Q2 What is your job title? Please state. For example, Screening and Immunisation Manager, Practice Nurse, Director of Public Health. If you have more than one job, please add others below.

____

Q2a If you have more than one job within the health sector, please state your other job title(s) here. For example, CCG Practice Nurse Lead, CCG Clinical Lead for Immunisation.

____

Q3 What organisation do you work for in the job that includes responsibility for managing the delivery of the immunisation programme? Please click the box that is most relevant for you

- ❍ Screening & Immunisation Team (PHE)/ Public Health Commissioners (NHS England) (1)
- ❍ Local Authority Public Health Team (2)
- ❍ Clinical Commissioning Group (3)
- ❍ Health Protection Team (Public Health England) (4)
- ❍ Immunisation provider organisation (5)
- ❍ Other (6)

Answer 3a) if ‘What organisation do you work for in the job that includes responsibility for managing the delivery of the immunisation programme? Immunisation provider organisation is selected

Q3a Please describe what type of provider organisation you work for. For example, GP Practice, NHS Hospital or Community Trust, social or private enterprise providing community health care services. Do not name you organisation.

Answer Q 3b, if 'What organisation do you work for in the job that includes responsibility for managing the delivery of the immunisation programme? Other is selected

Q3b If other, please state.

____

Section 2: Individual and organisational responsibility for Immunisation
In this section you will be asked some questions about your individual responsibilities for immunisation and the role of your organisation in managing and delivering the immunisation programme.

Q4 What are your primary individual responsibilities for the immunisation programme? Please list your top 2 or 3 priorities.

Highest priority (1)
Second highest priority (2)
Third highest priority (3)

Q4a Which of these responsibilities do you find the most challenging and why? Please comment here.

____
____
____
____
____

Q5 Which organisation is responsible for the overall leadership of the immunisation programme in your area?

- Health Protection Team (Public Health England) (1)
- Screening & Immunisation Team(PHE)/Public Health Commissioners (NHS England) (2)
- Local Authority Public Health Teams (3)
- Clinical Commissioning Groups (4)
- Not sure (5)

Q5a Please use this space for comments, if you are not sure, or want to say more about this.

____
____
____
____
____

Q6 How clear is the split of roles and responsibilities for immunisation between organisations in your area?

	Very clear (1)	Fairly Clear (2)	Not very clear (3)	Not clear at all (4)	Not sure (5)
The organisation responsible for commissioning the immunisation programme (1)					
The organisation responsible for training immunisation providers (2)					
The organisation responsible for monitoring and evaluating the quality of service providers' immunisation activities (6)					
The distribution of roles and responsibilities for immunisation across different organisations (9)					

Q60 Please add any comments you have about the distribution of roles and responsibilities across different organisations here.

____
____
____
____
____

Section 3: Working with others to manage the immunisation programme

In this section you will be asked some questions about how you work with other organisations to manage and the deliver the immunisation programme. For the purpose of this questionnaire we define partnership as ‘a mutually beneficial process by which two or more stakeholders or organisations work together to achieve a common goal’. Accordingly, partnership involves the joint development of structures in which decisions are made, resources shared and mutual authority and accountability exercised.

Q7 Who do you work with in order to fulfill your responsibilities for immunisation? Please click all boxes that are relevant

- ❑ Screening & Immunisation Teams (PHE)/ Public Health Commissioners (NHS England) (1)
- ❑ Clinical Commissioning Groups (2)
- ❑ Health Protection Team (Public Health England) (3)
- ❑ Local Authority Public Health Team (4)
- ❑ Schools (6)
- ❑ Maternity units (7)
- ❑ GP Practices (8)
- ❑ NHS hospital or community trusts (9)
- ❑ Social health care enterprises (10)
- ❑ Private health care enterprises (11)
- ❑ Others, please state below (12)

Answer Q7a if ‘Who do you work with in order to fulfill your responsibilities for immunisation?’ Others, please state below is Selected

Q7a If you clicked others, please state who here

Answer Q8, if ‘What organisation do you work for in the job that includes responsibility for managing, assuring...?’ Screening & Immunisation Team (NHS England/Public Health England) is selected

Q8 How many CCGs, Local Authorities Public Health Teams are there in your area? Please do not state any names just tell us how many.

Answer Q8a, if ‘What organisation do you work for in the job that includes responsibility for managing, assuring...?’ Local Authority Public Health Team is selected

Q8a What type of council do you work for?

- ❍ County (1)
- ❍ District (2)
- ❍ Unitary (3)
- ❍ London or other Metropolitan (4)
- ❍ Other (5)

Answer Q8ai, if ‘What type of council do you work for?’ Other is selected

Q8ai If you click other, please state what that is here.

Answer Q8b, if ‘What organisation do you work for in the job that includes responsibility for managing, assuring the immunisation programme?’ Clinical Commissioning Group is selected

Q8b How many GP Practices does your CCG include? Please do not state any names just provide us with information about the size of your area/number of practices your CCG covers.

____

Q9 How often are you involved in the following with reference to immunisation?

	Less than quarterly (1)	Quarterly (2)	Monthly (3)	Weekly (4)	Not applicable (5)
Attend the Local Authority Health Protection Forum (1)					
Attend an Immunisation Committee involving different partners in my area (2)					
Attend CCG immunisation meetings (e.g. CCG led Flu meetings) (4)					
Attend Local Authority Public Health Team Meetings (5)					
Attend Health & Wellbeing Board Meetings (6)					
Email/phone Local Authority Partners (7)					
Email/phone CCG Partners (8)					
Email/phone members of the Screening & Immunisation Team (9)					
Email/phone public health commissioners (10)					
Email/phone members of the Health Protection Team (11)					

Q10 Please list any other forums, means of working together that you use and how frequently.

____
____
____
____
____

Q11a If you attend an immunisation committee, what are its' aims and objectives? Please do not provide the name of the committee just state its' purpose.

Q11b If you attend an immunisation committee, please state what organisations are represented on this committee and which type of organisation chairs it (e.g. Local Authority Public Health Team, CCG, Screening and Immunisation Team). Please do not provide specific names of the organisations involved just what type of organisation they are.

Q14 Are you aware of any financial incentives for people to work in partnership to deliver the immunisation programme in your area?

- ❍ Yes (1)
- ❍ No (2)

Answer Q53, if 'Are you aware of any financial incentives for people to work in partnership to deliver the immunisation programme?' Yes is Selected

Q53 If you stated yes for financial incentives for partnership working, please state what these are.

Q15 Thinking about partnership working arrangements (e.g. immunisation committees and health protection forums) in your area, please state how strongly you think these help to support the following activities.

	Very Strongly (1)	Fairly Strongly (2)	Not very strongly (3)	Not at all strongly (4)	Not sure (5)
Information sharing about vaccination programmes and related activities (1)					
Sharing provider immunisation uptake data (2)					
Sharing expertise across organisations (3)					
Organising training and support for immunisation providers (4)					
Understanding local barriers to increasing immunisation uptake (5)					
Joint planning on how to increase vaccination coverage (6)					
Agreeing local priorities, setting targets and delegating responsibilities (7)					
Increasing access to immunisation services for people living in your area (9)					
Clarifying					

different organisations' roles and responsibilities (10)<br>Addressing poor performance at provider level (11)<br>Local authority scrutiny of immunisation performance (13)					

Q16 To what extent do you agree or disagree with the following statements about partnership working in your area.

	Strongly agree (1)	Agree (2)	Disagree (3)	Strongly disagree (4)	Not sure (5)
Partnership working improves the organisation of the immunisation programme in my area (1)					
Partnership working improves the performance of the immunisation programme in my area (2)					

Q17 What do you think are the key factors that are facilitating partnership working in your area? Please state up to three

1. (1)
2. (2)
3. (3)

Q18 The following are potential barriers to partnership working. Please indicate the extent to which any of these are currently a barrier in your area.

	Very significant barrier (1)	Fairly significant barrier (2)	Not a barrier (3)	Not sure (4)
The number of partner organisations involved (1)				
The geographic size of area covered (2)				
A lack of pre-existing immunisation partnership structures (pre 2013) (3)				
A lack of trust and confidence between organisations (4)				
The different cultures of the partner organisations (5)				
A lack of commitment from one or more partner organisations (7)				
A lack of strategic leadership (8)				
Dominance of one organisation in discussions (9)				
A lack of a shared vision and related action plan (10)				
A lack of shared ownership for agreed actions, thinking that one organisation is overall responsible (11)				
Non-overlapping				

geographic boundaries of partnership organisations (12)<br>Lack of provider representation in partnership working groups (13)<br>Lack of a budget to support actions taken as part of partnership working (14)				

Q19 Please state any other current operational barriers to partnership working in your area.

Q20 Do you think you would benefit from additional training in partnership working?

- Yes (1)
- No (2)
- Not sure (3)

Q21 What arrangements are in place in your area for sharing immunisation data (e.g practice level data) between partners? (Please tick the boxes that are relevant in your area)

- ❑ Data sharing agreement (1)
- ❑ Data shared with proviso that partners will keep it confidential (2)
- ❑ The data is not available to my organisation (3)
- ❑ The data cannot be shared (4)
- ❑ None (5)
- ❑ I do not know (6)
- ❑ Other, please state below. (7)

Q21a If you clicked other, please state what that is here

____

Section 4: Evaluating programme uptake and supporting immunisation providers
In this section you will be asked some questions about what is done to improve vaccination uptake in your area and how immunisation providers are supported and evaluated.

Q22 Who is involved in supporting immunisation providers in your area? (e.g. providing training, mentoring or practical advice)

	Very involved (1)	Quite involved (2)	Less involved (3)	Not involved (4)
Screening & Immunisation Teams (PHE)/ Public Health Commissioners (NHS England) (1)				
Local Authority Public Health Teams (2)				
Clinical Commissioning Groups (3)				
Health Protection Teams (PHE) (4)				

Q23 Who is involved in evaluating the performance of immunisation providers in your area?

	Very involved (1)	Quite involved (2)	Less involved (3)	Not involved (4)
Screening & Immunisation Teams (PHE)/ Public Health Commissioners (NHS England) (1)				
Local Authority Public Health Teams (2)				
Clinical Commissioning Groups (3)				
Health Protection Teams (PHE) (4)				

Q24 Who is involved in taking action to improve the performance of immunisation providers in your area?

	Very involved (1)	Quite involved (2)	Less involved (3)	Not involved (4)
Screening & Immunisation Teams (PHE)/ Public Health Commissioners (NHS England) (1)				
Local Authority Public Health Teams (2)				
Clinical Commissioning Groups (3)				
Health Protection Teams (PHE) (4)				

Answer Q25, if 'What organisation do you work for in the job that includes responsibility for managing the delivery programme? Immunisation provider organization Is Not Displayed

Q25 What does your organisation do to support immunisation providers in your area?

____
____
____
____
____

Q27 What does your organisation do to improve the quality of immunisation services in your area?

Answer Q61, if 'What organisation do you work for in the job that includes responsibility for managing the delivery of the immunisation programme?' Immunisation provider organisation is selected

Q61 What support do you receive to help you deliver the immunisation programme effectively (e.g. training, advice lines)? Who provides this support?

____
____
____
____
____

Answer Q28 if ‘What organisation do you work for in the job that includes responsibility for managing, assuring the immunisation programme? Clinical Commissioning Group is selected

Q28 What does your CCG do to evaluate the immunisation performance of constituent GP Practices?

____
____
____
____
____

Q29 Are any of the following done to support immunisation providers in your area?

	Yes (1)	No (2)	Not sure (3)
Provider visits by Screening and Immunisation Team members or Public Health Commissioners (1)	❍	❍	❍
Practice visits by CCG members (e.g. practice nurse leads) (2)	❍	❍	❍
Providers given access to foundation immunisation training (3)	❍	❍	❍
Providers given access to annual immunisation training updates (4)	❍	❍	❍
Providers given access to an immunisation advice telephone service (5)	❍	❍	❍
Providers given access to an immunisation advice email service (6)	❍	❍	❍
Best practice sharing workshops or similar forums (7)	❍	❍	❍

Q30 What else is done to support immunisation providers in your area? If you are not aware of anything else please state nothing.

____
____
____
____
____

Q31 To what extent do you think that what is done to support immunisation providers in your area is adequate?

- ❍ Very adequate (1)
- ❍ Adequate (2)
- ❍ Not adequate (3)
- ❍ Not at all adequate (4)
- ❍ Not sure (5)

Q32 Are any of the following done to help improve immunisation providers performance in your area?

	Yes (1)	No (2)	Not sure (3)
Circulation of reports that summarise provider performance (1)	❍	❍	❍
Peer review of immunisation uptake at CCG forums (3)	❍	❍	❍
Poorly performing providers are visited by members of the Screening and Immunisation Team (4)	❍	❍	❍
Provider visits by NHS England patient safety/quality team (5)	❍	❍	❍
Poorly performing practices are visited by members of CCG quality improvement team (6)	❍	❍	❍
Primary care commissioning team issue breach of contract warnings (7)	❍	❍	❍
Immunisation contracts are withdrawn and transferred to other providers (8)	❍	❍	❍

Q33 What else is done to improve immunisation providers performance in your area? If you are not aware of anything else, state nothing.

Q34 To what extent do you think that what is done to improve immunisation provider's performance in your area is adequate?

- ❍ Very adequate (1)
- ❍ Adequate (2)
- ❍ Not adequate (3)
- ❍ Not at all adequate (4)
- ❍ Not sure (5)

Q35 What else do you think could be done to support providers and improve immunisation performance in your area?

Conclusion

Finally, we would like you to reflect on how well the system for managing the immunisation programme is working in your area, and invite you to comment on any other factors that may be having an impact on the way the immunisation programme is managed and delivered both in your area and nationally.

Q36 Please indicate how confident you feel about the following statements.

	Very confident (1)	Fairly confident (2)	Not very confident (3)	Not at all confident (4)	Prefer not to say (5)
I have confidence in the system for delivering the immunisation programme in my area (1)					
I have confidence that immunisation services in my area are delivered equitably (2)					
I have confidence in the way that inequalities in performance are monitored and addressed in my area (3)					

Q37 What do you think could be done to improve the way that the immunisation programme is organised and delivered in your area?

____
____
____
____
____
____
____
____
____
____

Q38 Please use this space to add any other comments either about the topics that were covered in this questionnaire or any other issues related to the immunisation programme.

____
____
____
____
____
____
____
____
____
____

Thank you very much for finishing the questionnaire. We are very grateful for your contribution to this survey.

As stated, all data will be anonymised in any reporting. If you have any questions or queries, do please contact us.

Yours sincerely,

Tracey Chantler
Research Fellow
LSHTM, 15-17 Tavistock Place,
London, WC1H 9SH
tracey.chantler@lshtm.ac.uk
IMMS_Survey@lshtm.ac.uk

Sandra Mounier- Jack
Senior Lecturer
LSHTM, 15-17 Tavistock Place
London, WC1H 9SH I
MMS_Survey@lshtm.ac.uk
