# Supplementary material for: Is partnership the answer? Delivering the national immunisation programme in the new English health system: a mixed methods study
Source: BMC Public Health. 2019 Jan 17;19:83. doi: 10.1186/s12889-019-6400-6 (PMC6337826; doi:10.1186/s12889-019-6400-6)
Supplement: Supplementary file 2 — Topics covered in the interviews in the qualitative evaluation (PDF 259 kb) [file 12889_2019_6400_MOESM2_ESM.pdf]

Topic areas covered in the interviews for the qualitative evaluation

- **Review of LIB experience**
  - Purpose of the board
  - Membership of the board
    - Representative of all stakeholders?
  - Governance of the board
  - What the board has achieved
  - What still needs to be done
  - What improvements could be made
  - Maintaining interest and momentum

- **Objectives of the board**
  - Clarity of objectives
  - Focus of the objectives
  - Ability to change and adapt as needed
  - Management of risks

- **Responsiveness of the board**
  - Ability to respond to problems/early warning signs
  - Communication and meeting schedules
  - Ability to take action

- **Sub-groups**
  - Purpose of sub-groups
  - Function of sub-groups
  - Achievements of sub-groups
  - Future of sub-groups

- **Partnership functioning**
  - Support of pan London collaboration
  - Promotion of collective responsibility for the aims of the immunisation programme
  - Links with national level boards and organisations
  - Links between LIB and groups leading on immunisation borough action plans

- **Forward planning**
  - Reviewing future of the board
    - Aims & objectives
    - Membership
  - Future focus
